# Clinical Manifestations in the Maxillofacial Region of Acute Crisis of Sickle Cell Anemia: A Case Report

**DOI:** 10.4317/jced.62933

**Published:** 2025-07-01

**Authors:** Martín Andura-Correas, Guillermo Chacón-Ferrer, Eduardo Vázquez-Salgueiro, Luis Ortiz-Peces, Álvaro Damián Moreiras-Sánchez, Jorge Noguera-Tomás, José Luis Cebrián-Carretero

**Affiliations:** 1 Oral and Maxillofacial Surgery Department, Hospital Universitario La Paz, Madrid, Spain (Paseo De La Castellana, 261, Madrid)

## Abstract

Sickle cell anemia, or sickle cell disease, is a hematologic condition commonly found in populations from the Middle East, India, the Caribbean, and the Mediterranean. One of its complications is the development of long bone osteomyelitis. However, mandibular bone involvement is rare, which makes early diagnosis of this disease difficult when it affects this anatomical region. In this article, we present the case of a 25-year-old male with a prior diagnosis of sickle cell anemia who developed mandibular osteomyelitis associated with a paramandibular abscess and ipsilateral temporomandibular joint involvement, which was diagnosed and treated medically and surgically. Through this case, we aim to provide clinical insight into mandibular infectious complications associated with sickle cell anemia, along with their diagnosis and therapeutic management. Multidisciplinary care, clinical evaluation, laboratory findings, and imaging studies are the main pillars for the management of this disease. Furthermore, we review the literature on the clinical presentation, diagnosis, and treatment of mandibular osteomyelitis in patients with sickle cell disease.

** Key words:**Sickle cell anemia, mandibular osteomyelitis, Vascular Occlusion Crisis.

## Introduction

Sickle cell anemia (SCA) is the most common hereditary hematological disease, affecting over 250 million people worldwide. These patients experience hemolytic anemia and microvascular occlusion leading to VOCs. They are at increased risk of osteoarticular complications such as osteomyelitis, septic arthritis, and osteonecrosis. Osteomyelitis is one of the most serious and potentially disabling manifestations.

The disease follows an autosomal recessive inheritance pattern due to a point mutation in the beta-globin subunit gene on chromosome 11. This results in substitution of glutamic acid with valine, leading to the production of hemoglobin S, which tends to polymerize in its deoxygenated state. This causes red blood cells to assume a crescent or “sickle” shape. Unlike normal erythrocytes, sickled cells have a markedly shortened lifespan and, due to their rigid structure, are more prone to become trapped in slow-flow venules.

The sickle cell trait occurs in heterozygous carriers of the mutation, who are relatively protected against malaria-related death caused by Plasmodium falciparum. This explains the high gene prevalence in malaria-endemic areas. However, carriers of the sickle cell trait usually do not develop symptoms.

From an oral and maxillofacial perspective, SCD presents several important considerations. Vaso-occlusive episodes can affect the maxillofacial region, potentially leading to osteomyelitis of the jaws, particularly the mandible, due to compromised vascular supply. Radiographic findings may include trabecular pattern changes or a “step-ladder” appearance in the alveolar bone. Furthermore, patients with SCD may have delayed tooth eruption, enamel hypoplasia, and increased susceptibility to infections. Dental and surgical procedures require careful planning to minimise stress, ensure adequate oxygenation, and avoid triggers of sickling. Multidisciplinary collaboration is essential for safe and effective care (1).

Given the global burden of SCD and its systemic implications, including those in the maxillofacial region, practitioners must remain vigilant in identifying potential complications and adapting treatment protocols accordingly. Preventative dental care, early intervention, and coordinated management are crucial in improving outcomes for affected individuals (2).

## Case Report

A 25-year-old male with a history of SCA presented to the Emergency Department at La Paz University Hospital due to a three-week course of vaso-occlusive crisis (VOC), characterized by pain in the lower limbs and jaw. The patient had good disease control with medical treatment, experiencing 2-3 mild VOC episodes per year. Upon arrival, he was hemodynamically sTable, afebrile, with no trismus or floor-of-mouth swelling, no dyspnea or dysphagia, and only mild right paramandibular swelling, soft on palpation, without dental mobility or percussion pain. Lab results showed acute renal failure parameters and leukocytosis, but acute phase reactants were within the normal range. The Hematology Department indicated hospital admission for VOC management with analgesics and empirical antibiotic therapy (amoxicillin-clavulanic acid). The patient showed clinical improvement in the first 48 hours. However, two days after admission, clinical worsening occurred with increased paramandibular swelling extending to the ipsilateral preauricular region and development of trismus with a 10 mm mouth opening. In the oral cavity, at the lingual cortex adjacent to the molars in the fourth quadrant, a small area of bone exposure was observed with purulent discharge.

After consulting the Internal Medicine Department, the antibiotic therapy was switched to piperacillin-tazobactam.

**Figure 1:**
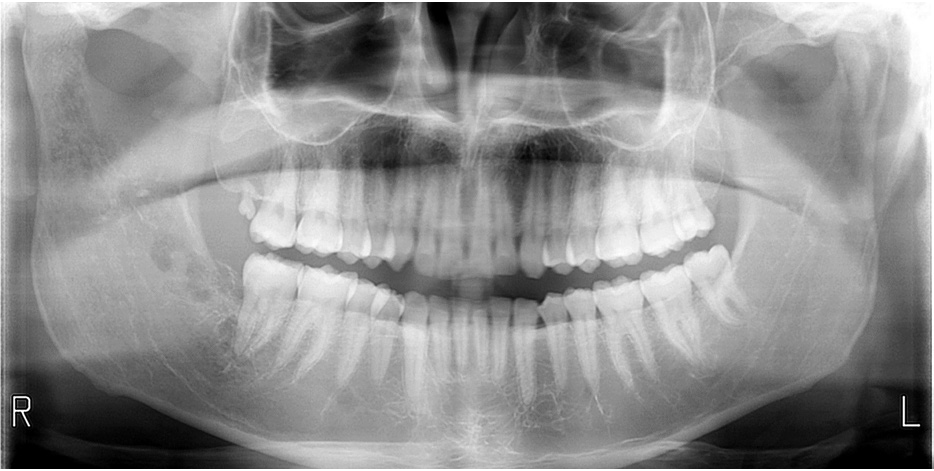
Orthopantomography.

**Figure 2:**
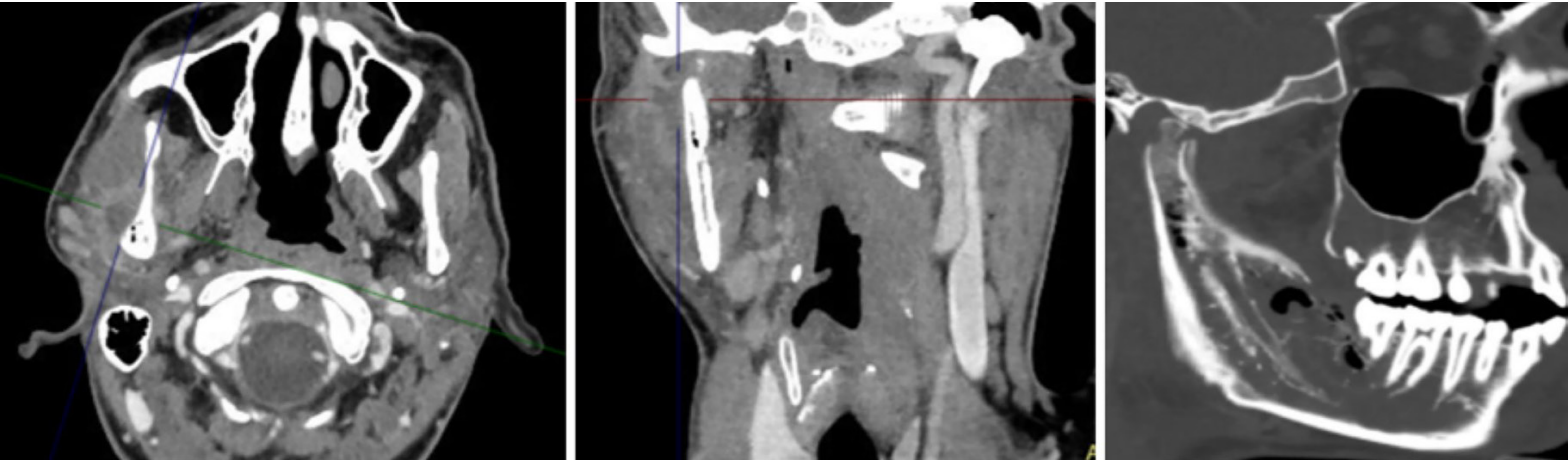
Contrast-enhanced CT scan.

An orthopantomography (Fig. 1) and contrast-enhanced CT scan (Fig. 2) revealed alteration of the right hemimandible involving the retromolar region of the mandibular body and the ascending ramus extending to the mandibular condyle. The affected area showed a permeative pattern with focal thinning and cortical destruction, periosteal reaction around the condyle and mandibular ramus, and multiple gas bubbles in the bone marrow of the affected angle and ramus—findings suggestive of right hemimandibular osteomyelitis. Additionally, signs were consistent with infectious arthritis of the right temporomandibular joint and an abscessed collection in the masticator space adjacent to the outer cortex of the ramus.

Surgical intervention was performed via an intraoral approach in the right retromolar trigone region, with drainage of purulent material and curettage of the sequestrum, from which samples were obtained for microbiological and histopathological studies.

The patient had a favorable postoperative course with improvement in trismus and pain.

One week later, under local anesthesia, the exposed bone sequestrum in the fourth quadrant (lingual cortex) was removed, and platelet-rich fibrin (PRF) was applied.

The culture of purulent material from the abscess revealed Streptococcus constellatus, sensitive to penicillin. In consultation with Internal Medicine Department, piperacillin-tazobactam was discontinued, and antibiotic treatment was completed with amoxicillin-clavulanic acid for a total of two weeks post-surgery.

No other crisis were developed in the following year, with periodic medical check-ups each month.

## Discussion

Haemoglobinopathies are a diverse group of inherited blood disorders that affect the structure or synthesis of haemoglobin, the oxygen-carrying component of red blood cells. They are typically inherited in an autosomal recessive pattern and include conditions such as thalassaemia and sickle cell disease (SCD). SCD is one of the most clinically significant and globally prevalent haemoglobinopathies, as well as one of the most common monogenic disorders worldwide (3).

Historically, haemoglobinopathies were geographically localized, primarily affecting populations in Africa, the Mediterranean basin, the Middle East, Southeast Asia, and the Indian subcontinent. However, due to increased global migration, these conditions now affect a far broader demographic. It is estimated that approximately 5.2% of the world’s population carries a significant haemoglobin variant, with over 332,000 births affected annually. Of these, more than 275,000 are cases of SCD, highlighting the importance of early diagnosis and preventative care.

Sickle cell disease encompasses a group of inherited red cell disorders characterised by the presence of haemoglobin S (HbS), a structurally abnormal variant of haemoglobin. This mutation arises from a single nucleotide substitution (GAG → GTG) in the sixth codon of exon 1 of the β-globin gene on chromosome 11, resulting in the substitution of valine for glutamic acid at position 6 of the β-globin chain. The abnormal HbS polymerises under deoxygenated conditions, distorting red blood cells from their typical biconcave shape into rigid, sickle-like forms. These distorted cells are prone to haemolysis and vaso-occlusion, leading to acute complications and long-term organ damage (3).

The clinical spectrum of SCD is broad and varies depending on the specific genetic mutation. The homozygous form, sickle cell anaemia (HbSS), is the most severe, resulting from the inheritance of the sickle gene from both parents. Compound heterozygous states also exist, in which the sickle gene is co-inherited with other abnormal haemoglobins such as HbC, HbD, or HbO-Arab, resulting in HbSC, HbSD, or HbSO-Arab disease respectively. Additionally, co-inheritance with thalassaemia alleles gives rise to sickle β°-thalassaemia or sickle β°-thalassaemia.

Sickling of red cells is exacerbated by physiological stressors such as hypoxia, dehydration, acidosis, infection, extreme temperatures, and hormonal changes. These conditions precipitate vaso-occlusive events and other acute complications, including painful crises, acute chest syndrome, splenic sequestration, and aplastic crises. Over time, repeated episodes contribute to cumulative damage in multiple organ systems.

In sickle cell disease, abnormal erythrocytes can accumulate in the bloodstream, occluding the microvasculature, triggering inflammatory pathways and infarctions in multiple organs and systems, particularly the spleen, liver, excretory system, nervous system, and musculoskeletal system. Children with this disease develop splenic hypofunction due to repeated infarctions, compromising their immune response to encapsulated organisms. Combined with infarcted bone, this increases the risk of osteomyelitis.

In the general population, *Staphylococcus aureus* is the most common organism associated with osteomielitis (4). However, in the US, gram-negative enteric bacilli such as Salmonella spp. are more commonly found in sickle cell patients (70%) compared to S. aureus (16.4%). It is hypothesized that these enteric bacteria arise from intestinal ischemic infarctions during VOC episodes (5).

In the acute phase, VOC and osteomyelitis are almost indistinguishable, although the former is more common. Both may present with fever, localized inflammation, and painful limitation of joint mobility. Osteomyelitis typically affects long bone diaphyses, but other sites may also be involved. Fever and pain usually precede the acute presentation by at least 24 hours. Osteomyelitis patients are less likely to have multiple tender points than those with VOC (6).

Lab tests are inconclusive for differentiating mandibular osteomyelitis (MO) from VOC, as both can present with leukocytosis and elevated inflammatory markers (CRP and ESR). Differentiation is challenging without a positive bacterial culture.

The gold standard for diagnosing osteomyelitis is bacteriological confirmation from bone, synovial fluid, or blood cultures. However, their absence does not rule out the diagnosis. The invasive nature of obtaining organic samples and the relative frequency of false-negative blood cultures makes imaging crucial for identifying the condition.

Clinical features, lab findings, and imaging studies must be complementary to establish a diagnosis, guide therapy, and estimate prognosis.

Initial medical management of an acute crisis includes intravenous hydration, oxygenation, and pain control. Ideally, appropriate cultures should be obtained before administering antibiotics to increase the chances of pathogen identification—except in cases of sepsis or hemodynamic instability. If MO is ruled out, empirical antibiotics may be discontinued.

The accepted antibiotic duration for MO is 4–6 weeks. Antibiotic selection should ideally be based on microbiological results from collected samples. According to the 2019 Cochrane review, there is limited literature to guide antibiotic selection for MO in SCA patients, highlighting the importance of multidisciplinary management involving Microbiology, Infectious Diseases, and Pathology services (5).

Most reported cases of MO in SCA patients involve surgical debridement or sequestrectomy and targeted antibiotics. However, we advocate for antimicrobial therapy as the mainstay, reserving surgery for diagnostic confirmation, abscess drainage, or removal of necrotic bone (7).

## Conclusions

Sickle cell disease increases the risk of osteoarticular complications, such as osteomyelitis.

Although the mandible is an uncommon site for MO in SCA, it should be considered as a possible location.

Multidisciplinary care, combining clinical evaluation, laboratory findings, and imaging studies, is essential for accurate diagnosis and proper therapy to reduce potential complications.

## Data Availability

The datasets used and/or analyzed during the current study are available from the corresponding author.
